# Detection of carrier Booroola (*Fec*^B^) allele in BMPR1B gene of MEGA (Merino × Garut) sheep and its association with growth traits

**DOI:** 10.1186/s43141-023-00475-z

**Published:** 2023-02-15

**Authors:** Endang Tri Margawati, Widya Pintaka Bayu Putra, Muhammad Rizki, Edi Soetrisno, Herman Willem Raadsma

**Affiliations:** 1 Research Center for Applied Zoology, National Research and Innovation Agency (BRIN), Bogor, 16911 Indonesia; 2 Department of Animal Science, Faculty of Agriculture, University of Bengkulu, Bengkulu, 38371 Indonesia; 3 Center for Advanced Technologies for Animal Genetics and Reproduction, Faculty of Veterinary Science, University of Sydney, Camden, NSW 2006 Australia

**Keywords:** BMPR1B gene, Boorola, Growth traits, MEGA sheep, PCR–RFLP

## Abstract

**Background:**

Bone morphogenetic protein receptor 1B (BMPR1B) gene is one of candidate genes for reproductive and growth traits in sheep. The present study was aimed to detect the Booroola (*Fec*^B^) allele in BMPR1B gene and its association with growth traits in MEGA (Merino × Garut) sheep. A total of 82DNA samples collected from individual lamb (mixed-sex) blood were genotyped for allelic polymorphism using a PCR–RFLP method.

**Results:**

The PCR analysis in BMPR1B gene resulted the amplicons with size of140 bp. The RFLP analysis with *Ava*II restriction enzymeresultedtwo allelic types of wildtype (A/*Fec*^+^) and mutant or Booroola (G/*Fec*^B^) with frequency of 0.89 and 0.11, respectively. However, the genetic diversity in BMPR1B/*Ava*II gene of animal studies was categorized tolow category (PIC = 0.18)and under in a genetic equilibrium (*χ*^2^ = 1.25).

**Conclusions:**

Itshowed us that carrying *Fec*^B^ allele in the heterozygous sheep were not associated with growth traits in MEGA sheep.

## Background

Bone morphogenetic protein receptor 1B (BMPR1B) gene is one of the candidate genes for prolificacy trait in sheep (Juengel et al. 2013) [1]. The BMPR1B gene with a coding sequence 17 exons has been mapped on sheep chromosome 6 along 451,922 bp (GenBank: NC_056059.1). A transitional mutation (A to G) has been occured in the exon 8 of *ovine* BMPR1B gene namely Booroola (*Fec*^B^) mutation (Kumar et al. 2021) [2]. In addition, the *Fec*^B^ mutation was occured at 109^th^ nucleotide (GenBank:GQ863576.1)with an amino acid changes from Glutamine (Q) to Arginine (R).This mutation is located in the kinase highly conserved domain BMPR1B or activin-like kinase 6 (ALK6) and characterized by precocious differentiation of ovarian follicles, leading to the production of large members of ovulatory follicles that are smaller in diameter than wildtype follicles (Souza et al. 2003) [3].

Interestingly, the *Fec*^B^ mutation also affected to themany productive traits.A previous studies reported that the *Fec*^B^ mutation was significantly associated with ovulation rate (Davis, 2005) [4], litter size (Mahdevi et al. 2014 [5]; Maskur et al. 2016) [6], body weight (Gootwine et al. 2006 [7]; Guan et al. 2007 [8]), wool production (Schulze et al. 2003) [9], lamb survival (Gootwine et al. 2008) [10] and carcass traits (Fahmy et al. 1992 [11]; Dimitrov and Nedelchev, 1999 [12]).

The *Fec*^B^ mutation was firstly detected in Booroola Merino sheep from Australia.Unfortunately, there are a few studies to detect the *Fec*^B^ mutation in Indonesian sheep. Maskur et al. (2016) [6] reported that the evidence of*Fec*^B^ mutation was observed in fat-tailed sheep breed and associated with litter size.Merino sheep has been imported in Indonesia to increase meat production through crossbreeding program with local ewes.Garut is one of Indonesian native thin-tailed sheep breeds that potential for crossbreeding program with Merino sheep. Previous studies reported that the average of adult weight in Garut rams was39.53 ± 1.95 kg (Rosmawan et al. 2021) [13] and carcass weight in Garut ewes was23.63 ± 2.39 kg (Prahasta, 2015) [14]. In addition, Haya et al. (2020) [15] reported Garut ewes at the breeding station has a type of birth single (34.87%), double/twin (45.16%), triple/triplet (19.26%), quadruple/quartet (0.51%) and pentuple (0.16%).

The cross breeding program between Merino and Garut sheep breeds is potential to produce a crossbred sheep with high meat production and litter size traits. Hence, the selection program in the crossbred sheep (Merino × Garut) is important to improve their productivity. This study was aimed to detect the evidence of Booroola (*Fec*^B^) mutation in the MEGA (Merino × Garut) sheep using a PCR–RFLP method. In addition, the present study was aimed to observe the effect of *Fec*^B^ mutationonthe growth traits of sheep.The results of this study can be used as the early information to select sheep based on BMPR1B gene polymorphism.

## Methods

### Animal and DNA extraction

A total of eighty two (82) mixed-sex MEGA (Merino × Garut) sheep kept at the research station (LIPI and BALITVET) of Bogor, West Java-Indonesia were used in this study. About 7–10 mL of blood sample was taken in each animal from *jugular vein*using a venoject needle with vacutainer vacum tube containing EDTA and held on ice box until delivery to the laboratory for further experiments. The genomic DNA was extracted using a modified method of Montgomery and Sise (1990) [16].

### PCR analysis

**Fig. 1:**
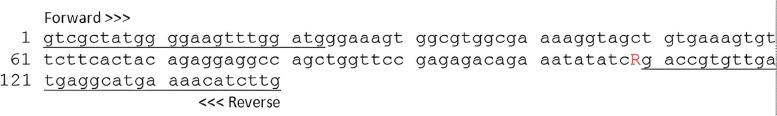
Primer position (underline) in the exon 8 of *ovine* BMPR1B gene (GenBank: GQ863576.1) along 140 bp. A Boorola (G or *Fec*.^B^) allele was caused by the missence mutation of c.109A > G or p.Q36R (R)

The PCR amplification of *ovine* BMPR1B gene was performed using a primer pair of Forward: 5′-GTC GCT ATG GGG AAG TTT GGA TG-3′ and Reverse: 5′-CAA GAT GTT TTC ATG CCT CAT CAA CAC GGT C-3′ (Wilson et al. 2001) [17]. According to that primer, the target sequence of BMPR1B gene is along 140 bp (Fig. 1). The PCR reaction was performed in a total volume 10 μL containing 1.2 μL of DNA template (2.18 ng/μL), 10 pmol/μL each of primer, 2 × of DreamTaq Green PCR mastermix kit (ThermoScientific, USA) and 3.6 μL of nuclease-free water. The PCR reaction was performed in a Mastercycler Gradient thermocycler (Eppendorf-Germany) with amplification program comprised of pre-denaturation (95 °C at 2 min); followed by 36 cycles of denaturation (95 °C at 30 s), annealing (56.6 °C at 1 min; 30 s), and extension (72 °C at 30 s); and final extension (72 °C at 2 min).

Electrophoresis of PCR product (amplicon) was performed using 1% agarose at 100 V at 30 min. Amplicons were stained with GelRed (Biotium, USA) along 30 min and then visualized using G-Box documentation system (Syngene, UK).

### RFLP analysis

For genotyping, amount of 10 μL of reaction solutions was used for RFLP analysis with containing of 2 μL PCR product, 0.2 μL of *Ava*II (G*GWCC) restriction enzyme (ThermoScientific, USA), 1 μL of enzyme buffers and 6.8 μL of nuclease-free water and placed in the water bath for 1 h at 37 °C for digestion. Digested samples were then quantified to visualize the amplified fragments by gel electrophoresis (2% agarose). After digestion with *Ava*II, a wild type animal (AA or *Fec*^+^/*Fec*^+^genotype) can be signedwith the presence of one DNA fragment with size of 140 bp in an agarose gel. A mutant animal (GG or *Fec*^B^/*Fec*^B^ genotype) signed with two DNA fragments along 109 bp and 31 bp. Meanwhile, the carrier animal (AG or *Fec*^+^/*Fec*^B^ genotype) signed with three DNA fragments of 140 bp, 109 bp, and 31 bp.

### Sequencing analysis

A forward sequencing analysis was performed with a carrier sample (30 μL of PCR product) to confirm the Booroola mutation site in the animal study. The sequencing analysis was performed by a commercial laboratoryservices (1st BASE Laboratories Sdn Bhd, Malaysia) with ABI Prism 96-capillary 3730 xl DNA Analyzer (Applied Biosystems, USA).

### Data analysis

The data of growth traits was collected from a herd book of year 1999 to 2002. The animal used in this study was born on first parity of dam.Hence, the data correction was performed in body weight to reduce the effect of sire, sex, and type of birth by using an equation as follows (Hardjosubroto, 1994) [18]:

$${\mathrm{BW}}_{\mathrm{c}} =\mathrm{ BW }\times {\mathrm{CF}}_{\mathrm{sex}} \times {C}_{\mathrm{TB}}$$

$${\mathrm{WW}}_{\mathrm{c}}=\left(\mathrm{BW}+\left(\frac{\mathrm{WW}-\mathrm{BW}}{{\mathrm{T}}_{\mathrm{w}}}\right)\times 120\right)\times {\mathrm{CF}}_{\mathrm{sex}}\times {\mathrm{CF}}_{\mathrm{TB}}$$

$${\mathrm{YW}}_{\mathrm{c}}=\left({\mathrm{WW}}_{\mathrm{c}}+\left(\frac{\mathrm{W}-{\mathrm{WW}}_{\mathrm{c}}}{\mathrm{T}}\right)\times 245\right)\times {\mathrm{CF}}_{\mathrm{sex}}\times {\mathrm{CF}}_{\mathrm{TB}}$$

$${\mathrm{DG}}_{\mathrm{pre}} = \left({\mathrm{WW}}_{\mathrm{c}}- {\mathrm{BW}}_{\mathrm{c}}\right)/120$$

$${\mathrm{DG}}_{\mathrm{post}} = \left({\mathrm{YW}}_{\mathrm{c}}- {\mathrm{WW}}_{\mathrm{c}}\right)/245$$

$${\mathrm{CF}}_{\mathrm{sex}} = {\mathrm{BW}}_{\mathrm{male}}/{\mathrm{BW}}_{\mathrm{female}}$$

where BW_c_ is the corrected birth weight; WW_c_ is the corrected weaning weight; YW_c_ is the corrected yearling weight; BW is the actual birth weight; WW is the actual weaning weight; *W* is the actual weight; *T*_w_ is the weaning age; *T* is the period beetwen weaning to weighing times; DG_pre_ is the pre-weaned daily gain; DG_post_ is the post-weaned daily gain;CF_sex_is the correction factor for sex; *C*_TB_ is the constanta for type of birth, i.e., 1.0 (single)and 1.10 (twin). Therefore, the statistical analyses of genotypic and allelic frequencies, observed heterozigosity (*H*_o_), expected heterozigosity (*H*_e_), number of effective allele (n_e_), polymorphic informative content (PIC), and chi-square (χ^2^) value were computed according to Yasuda (1988) [19] to evaluate the genetic diversity in the BMPR1B gene of animal studies.

## Results

**Fig. 2:**
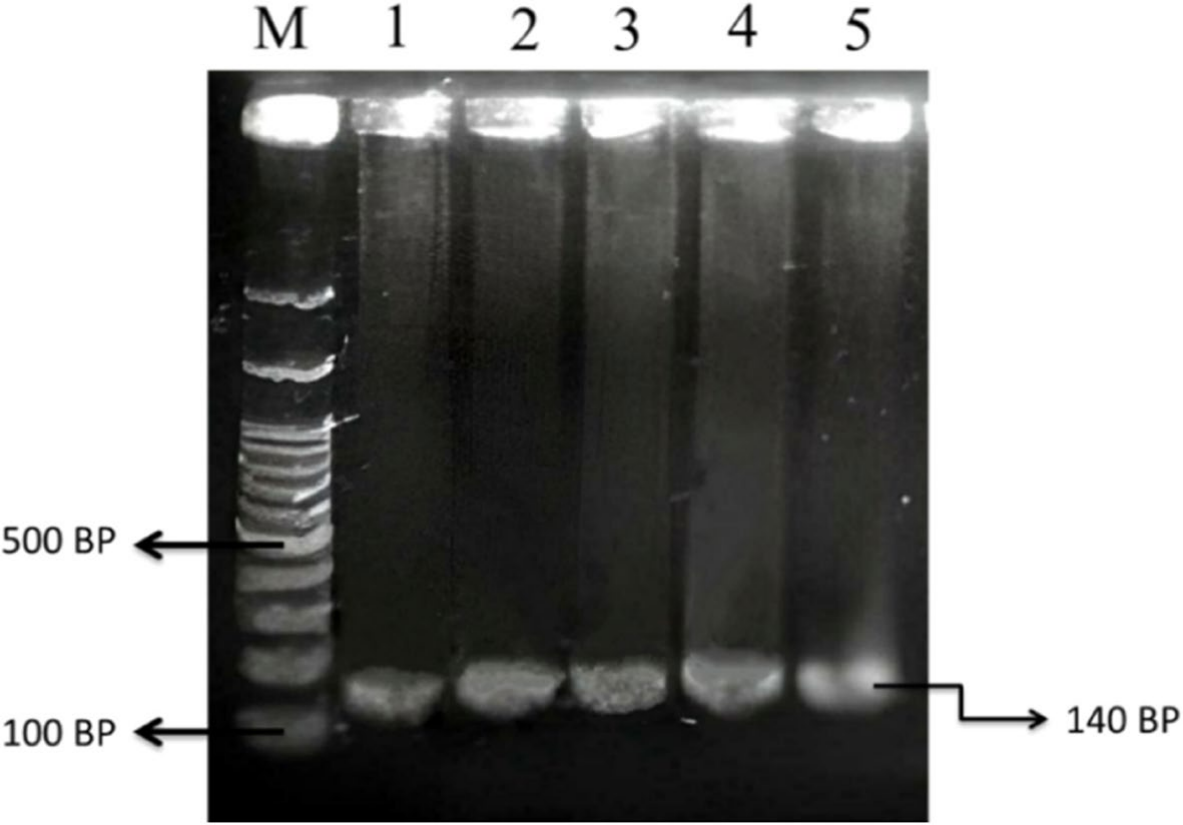
The PCR results for BMPR1B gene in MEGA sheep with presence of DNA fragment (amplicon) along 140 bp in the 1% of agarose gel. M: DNA marker 100 bp; Line 1–5: DNA samples

**Fig. 3:**
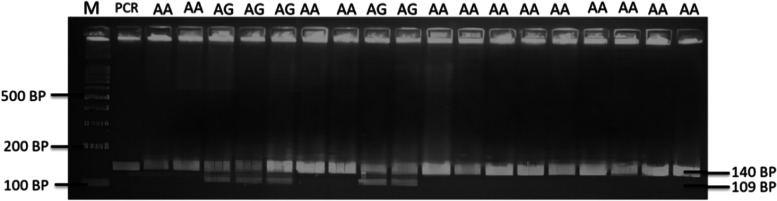
The PCR–RFLP results for BMPR1B/*Ava*II gene in MEGA sheep in 2% agarose gel were showed two genotype of AA-wildtype or *Fec*^+^/*Fec*^+^ (140 bp) and AG-carrier or *Fec*^+^/*Fec*^*B*^ (140 bp and 109 bp). PCR: amplicon (140 bp). M: DNA marker 100 bp

**Fig. 4:**
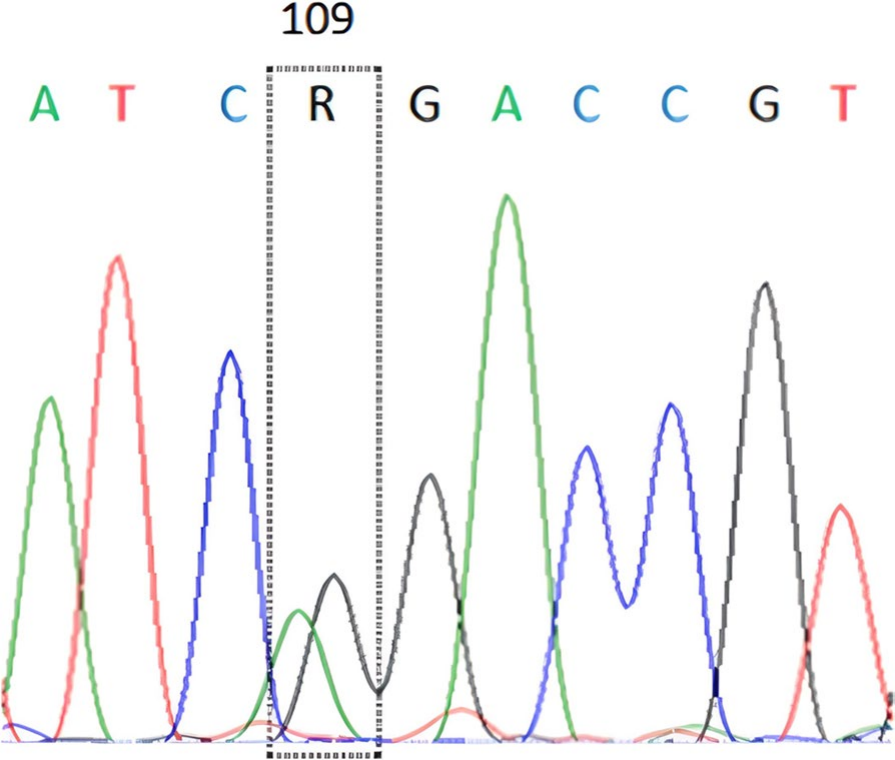
The Booroola mutation site (c.109A > G) in BMPR1B gene (exon 8) of MEGA sheep. R: A/G

The target gene of BMPR1B gene in animal studies was successfully amplified with signed by presence of a DNA fragment size of 140 bp in the 1% of agarose gel (Fig. 2). Therefore, the PCR–RFLP analysis in BMPR1B/*Ava*II gene reveals of two genotype of AA-wildtype (*Fec*^+^/*Fec*^+^) and AG-carrier (*Fec*^+^/*Fec*^B^) as shown in Fig. 3. In this study, a 31 bp of DNA fragment was not ilustrated in the 1% agarose gel because of low size. However, absence of 31 bp in this study was not influenced by the genotyping of BMPR1B/*Ava*II gene. Hence, a carrier sheep in this study signed with two DNA fragements along 140 bp and 109 bp. In addition, the presence of Booroola mutation site (c.109A > G) was confirmed in the carrier MEGA sheep as shown in Fig. 4. In addition, sheep with carrier *Fec*^B^ allele (heterozygous animal) was observed with low frequency (0.22) as presented in Table 1. Moreover, the frequency of *Fec*^B^ allele in animal studies was 0.11 and lower than *Fec*^+^ allele (0.89). However, the polymorphism of BMPR1B/*Ava*II gene was not associated with growth traits in MEGA sheep (Table 2). Interestingly, the MEGA sheep with heterozygote genotype (*Fec*^+^/*Fec*^B^) have the higher of birth weight, yearling weight and post-weaned daily gain values.

**Table 1:** The genetic diversity of BMPR1B/*Ava*II gene in MEGA sheep

Genotypic frequency (*N*)			Allelic frequency						
AA (*Fec*^+^/ *Fec*^+^)	AG (*Fec*^+^/*Fec*^B^)	GG (*Fec*^B^/*Fec*^B^)	A (*Fec*^+^)	G (*Fec*^B^)	*H*_o_	*H*_e_	*n*_e_	PIC	*χ*^2^
0.78 (64)	0.22 (18)	0.00 (0)	0.89	0.11	0.22	0.20	1.24	0.18	1.25*

*N* number of observation, *H*_*o*_ observed heterozigosity, *H*_*e*_ expected heterozygosity, *n*_*e*_ number of effective allele, *PIC* polymorphic informative content, *χ*^*2*^ chi-square value

^*^Under Hardy–Weinberg equilibrium

**Table 2:** Average growth traits in MEGA sheep based on the genotype of BMPR1B/*Ava*II gene

Growth traits^a^	Genotype (N)		*P* value
	AA (*Fec*^+^/*Fec*^+^)	AG (*Fec*^+^/*Fec*^B^)	
Birth weight (kg)	3.66 ± 0.59 (30)	3.90 ± 0.52 (5)	0.724
Weaning weight at 120 days of age (kg)	17.31 ± 3.23 (30)	16.81 ± 3.18 (5)	0.807
Yearling weight at 365 days of age (kg)	42.46 ± 17.29 (29)	48.10 ± 12.48 (5)	0.103
Pre-weaned daily gain (kg/day)	0.11 ± 0.03 (29)	0.10 ± 0.03 (5)	0.558
Post-weaned daily gain (kg/day)	0.10 ± 0.07 (29)	0.13 ± 0.06 (5)	0.250

*N* number of animal

^a^Corrected

## Discussion

Mostly the *Fec*^B^ allele was absence in many sheep breeds as shown in Table 3. However, low frequency of *Fec*^B^ allele has been reported in Indonesian Fat Tail (0.19) [6], Nilagiri (0.14) [20], Bayanbulak (0.08) [21], Kalehkoohi (0.35) [5] sheep (Table 3). In contrast, high frequency of *Fec*^B^ allele has been reported in Assaf (0.54) [7], Garole (0.61) [22], Bonpala (0.87) [23], Small Tail Han (0.72) [24], and Hu (0.84) [24] sheep (Table 3). However, the absence of *Fec*^B^ allele in sheep can be described as genetic drift evidence that caused by selection and migration factors [25]. Furthermore, the polymorphism in the BMPR1B/A*va*II gene of animal study belongs to low category, signed with low PIC value (PIC < 0.30). However, the chi-square (*χ*^2^) value revealed that the genetic diversity in BMPR1B/*Ava*II is under the genetic equilibrium (*χ*^2^ < 3.84).

**Table 3:** Allelic frequency in the BMPR1B/*Ava*II gene in many sheep breeds

Breed	Origin	*N*	Allelic frequency		Reference
			A (*Fec*^+^)	G (*Fec*^B^)	
Assaf	Israel	294	0.46	0.54	Gootwine et al. 2006[7]
Barbarine	North Africa	334	1.00	0.00	Borni et al. 2011[26]
Karakul de Botosani	Romania	20	1.00	0.00	Georgeseu et al. 2011[27]
Palas	Romania	60	1.00	0.00	Georgeseu et al. 2011[27]
Najdi	Saudi Arabia	69	1.00	0.00	Abouheif et al. 2011[28]
Naeimi	Saudi Arabia	55	1.00	0.00	Abouheif et al. 2011[28]
Blackbelly	Mexico	20	1.00	0.00	Lopez-Ramirez et al. 2014 [29]
Fat-Tailed	Indonesia	250	0.81	0.19	Maskur et al. 2016 [6]
Deccani	India	230	1.00	0.00	Pardeshi et al. 2005 [30]
Bannur	India	26	1.00	0.00	Pardeshi et al. 2005 [30]
Madras Red	India	20	1.00	0.00	Pardeshi et al. 2005 [30]
Garole	India	22	0.31	0.61	Polley et al. 2010 [22]
Bonpala	India	97	0.13	0.87	Roy et al. 2011 [23]
Nilagiri	India	145	0.86	0.14	Sudhakar et al. 2013 [20]
Small Tail Han	China	140	0.28	0.72	Chu et al. 2011 [24]
Hu	China	35	0.16	0.84	Chu et al. 2011 [24]
Texel	China	36	1.00	0.00	Chu et al. 2011 [24]
Chinese Merino	China	38	1.00	0.00	Chu et al. 2011 [24]
Bayanbulak	China	120	0.92	0.08	Zuo et al. 2013 [21]
Kalehkoohi	Iran	92	0.65	0.35	Mahdavi et al. 2014 [5]
Arabic	Iran	100	1.00	0.00	Mohammadi, 2016 [31]
Mehraban	Iran	115	1.00	0.00	Talebi et al. 2018 [32]
Hamdani	Iraq	64	1.00	0.00	Al-Barzinji, 2010 [33]
Awassi	Iraq	82	1.00	0.00	Sulaiman et al. 2014 [34]
Kurdi	Iraq	-	1.00	0.00	Al-Barzinji and Taha, 2017 [35]
Arabi	Iraq	-	1.00	0.00	Al-Barzinji and Taha, 2017 [35]
Awassi × Barki	Egypt	20	1.00	0.00	El-Hanafy and El-Saadani, 2009 [36]
Barki	Egypt	79	1.00	0.00	Ahmed et al. 2016 [37]; Othman et al. 2018 [38]
Ossimi	Egypt	28	1.00	0.00	Othman et al. 2018 [38]
Rahmani	Egypt	22	1.00	0.00	Othman et al. 2018 [38]
Saudanez	Egypt	38	1.00	0.00	Farag et al. 2018 [39]
Creole	Colombia	167	0.62	0.38	Hernandez et al. 2020 [40]
Watish	Sudan	156	1.00	0.00	Mohamed et al. 2020 [41]

*N* Number of animal

Low PIC value in the present study indicated that the BMPR1B/*Ava*II gene can not be used as molecular selection because of low genetic diversity. According to Table 2, the *Fec*^B^ (Booroola) allele included of a minor allele since the *Fec*^B^/*Fec*^B^ is typical of rare genotype. There are many factors causing the allelic frequency such as selection, migration, cross breeding and inbreeding Falconer DS (1996) [42].

The selection may be the main factor that affecting allelic frequency of *Fec*^B^ in MEGA since the farmers prefer to keep sheep with low litter size. According to the farmers expirience, the survival rate of single lambs are better than twin kids or triplet lambs. This statement supported by Sodiq [43] who reporting the significant effect between litter size with survival rate in sheep.

This preliminary study showed that the carrying *Fec*^B^ allele was not affected by the growth traits in MEGA sheep (Table 2). The similar finding reported by Abella et al. (2005) [44] that there was no effect of carrying *Fec*^B^ allele in the growth traits in Boorola × Merinos d’Arles sheep. However, present study reported that the average of BW, YW and DG_post_ in heterozygous sheep (*Fec*^+^/*Fec*^B^) were higer than those in wild type sheep(*Fec*^+^/*Fec*^+^). Prevous studies reported that *Fec*^+^/*Fec*^B^ genotype was as superior genotype for adult weight in Assaf sheep (Gootwine et al. 2006) [7] and yearling weight in Garole × Malpura sheep (Kumar et al. 2008) [45].

In the future, study to observe the effect of *Fec*^B^ mutation on reproductive traits of MEGA sheep is important for developing marker assisted selection (MAS). A previous studies reported that carrying *Fec*^B^ allele affected to the litter size of Indonesian Thin Tail (Maskur et al. 2016) [6], Mehraban (Talebi et al. 2018) [32], Kalehkoohi (Mahdavi et al. 2014) [5], Small Tail Han (Chu et al. 2011) [24] and Colombian Creole (Hernandez et al. 2020) [40]. In addition, a previous studies reported that two novel mutation of c.35 T/A and c.113A/G were detected in BMPR1B gene (GenBank: GQ863576.1) as reported by Farag et al. (2018) [39] and Talebi et al. (2018) [32], respectively. In this study, the evidence of mutation c.35 T/A was not detected with the forward sequencing. Meanwhile, the mutation c.113A/G did not occur in the MEGA sheep as shown in Fig. 4. Furthermore, the intronic region of BMP15 gene has the potency as the genetic markers for sheep since it has many insertion/deletion (indel) mutation sites [46]. Despite, a another *BMP* family genes of *BMP2* and *BMP7* are potential as the candidate genes for litter size of sheep (Li et al. 2021) [47].

## Conclusion

The carrier Booroola (*Fec*^B^) allele was detected in the MEGA (Merino × Garut) sheep with low frequency and not associated with the growth traits. However, birth weight, yearling weight and post-weaned daily gain in homozygous sheep (*Fec*^+^/*Fec*^B^) were higher than those in wildtype sheep (*Fec*^+^/*Fec*^+^).

## Data Availability

All data are primary data and generated from the research, research materials belong to our laboratory (Laboratory of Animal Molecular Genetics).

## Abbreviations

*Fec*^*B*^: Fecundity Boorola
BMPR 1B: Bone morphogenetic protein receptor 1B
MEGA: Crossing Merino × Garut sheep
PCR: Polymerase chain reaction
PCR-RFLP: Polymerase chain reaction- restriction fragment length polymorphism
ALK6: Activin-like kinase 6
EDTA: Ethyelene diamine tetra acetic acid
BW: Body weight
YW: Yearling weight
DG_*post*_: Post-weaned daily gain
